# Soybean induced resistance to *Spodoptera eridania* herbivory

**DOI:** 10.1002/ps.71029

**Published:** 2026-06-15

**Authors:** Bruno Henrique Sardinha de Souza, Arlindo Leal Boiça Júnior, Durvalina Maria Mathias dos Santos, Moacir Rossi Forim, Antonio Pizolato Neto, Andreísa Fabri Lima, Michael Joseph Stout

**Affiliations:** ^1^ Federal University of Lavras Lavras Brazil; ^2^ São Paulo State University Jaboticabal Brazil; ^3^ Federal University of São Carlos São Carlos Brazil; ^4^ Louisiana State University Baton Rouge LA USA

**Keywords:** antioxidant enzymes, flavonoids, IPM, proteinase inhibitors, southern armyworm

## Abstract

**BACKGROUND:**

Constitutive resistance is expressed constantly during plant development, whereas induced resistance can be triggered after herbivory. Evaluating induced resistance in soybean varieties and its correlation with expression of defense compounds and plant growth can aid in genotype screening and the use of induced resistance in integrated pest management. This study used two soybean varieties previously selected based on differing constitutive antibiosis‐resistance against *Spodoptera eridania* to test the hypotheses: prior herbivory by *S. eridania* induces higher resistance to conspecific larvae; resistance induction is associated with increased activities of defense proteins; induced resistance is related to increased flavonoid levels; and induced resistance trades off with plant growth.

**RESULTS:**

Effects of variety and injury were significant for leaf area consumed; injury affected larval survival; and variety × injury interacted for larval weight. The resistant variety showed constitutively higher superoxide dismutase, catalase, ascorbate peroxidase, polyphenol oxidase, and malondialdehyde levels. Herbivory decreased superoxide dismutase and polyphenol oxidase activities in both varieties and increased Kunitz trypsin inhibitors in the resistant variety. Isoquercitrin and daidzin were exclusive to the resistant and susceptible soybeans, respectively. Herbivory did not change flavonoid concentrations. Plant growth was not affected by herbivory, and susceptible plants grew faster than resistant soybeans.

**CONCLUSIONS:**

Evaluated enzymes and Kunitz trypsin inhibitors play a role in soybean constitutive resistance. Hydrogen peroxide is involved in injury signaling and malondialdehyde correlated with constitutive and induced resistance. Kunitz trypsin inhibitors increased after herbivory only in the resistant variety. Isoquercitrin is involved in constitutive resistance. There was no plant growth trade‐off with induced resistance. © 2026 The Author(s). *Pest Management Science* published by John Wiley & Sons Ltd on behalf of Society of Chemical Industry.

## Graphical abstract

SOD, CAT, APX, PPO, KTI, and MDA levels were higher in the resistant soybean. *S. eridania* herbivory increased H_2_O_2_ and MDA, but decreased SOD and PPO levels. *S. eridania* herbivory increased KTI activity only in the resistant variety. Isoquercitrin and daidzin were exclusive to resistant and susceptible soybeans. Soybean plant growth trades‐off with constitutive resistance.

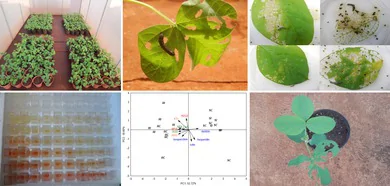

## 1 INTRODUCTION

Plants possess resistance mechanisms that protect them from the attack of insect pests, and these can be classified as constitutive and induced. Constitutive resistance is expressed somewhat constantly during plant development, whereas induced resistance can be manifested in plants after insect herbivory.^1^, ^2^, ^3^, ^4^, ^5^ Induced resistance has been reported in more than 100 plant species, including soybeans, *Glycine max* (L.) Merrill. The expression of induced resistance in soybeans can negatively affect the feeding and development of insect pests infesting the plants.^6^, ^7^, ^8^ It is assumed that induced resistance can save energy and resources that plants can invest only under stressful conditions, not compromising their growth and reproduction.^9^, ^10^, ^11^ Therefore, a trade‐off between plant defense and growth is expected.^2^, ^3^, ^4^

The Southern armyworm *Spodoptera eridania* (Cramer) (Lepidoptera: Noctuidae) is a polyphagous insect species that stands out as a pest of several crop plants with economic importance.^12^ In Brazil, *S. eridania* has gained increased prominence in soybean crops in the last two decades in function of frequent pest outbreaks, reaching population densities and defoliation levels higher than the estimated economic injury levels, being widespread in the major production regions.^13^, ^14^ The transgenic Bt soybean varieties currently available in the marketplace express the Cry1Ac, Cry1F, Cry1A.105, and Cry2Ab2 proteins that do not meet the high‐dose status for effective *S. eridania* control. In addition to being a voracious defoliator, later‐instar larvae can also attack soybean pods and seeds, directly impacting crop yield.^14^ Therefore, the screening of soybean germplasm for insect‐resistance traits is fundamental for developing commercial varieties resistant to *S. eridania*.

Understanding complex insect‐resistance mechanisms can aid in the development of protocols that use reliable chemical markers for high‐throughput phenotyping of soybean germplasm with induced‐resistance traits. For instance, hydrogen peroxide (H_2_O_2_) is an important reactive oxygen species (ROS) generated in stressed plants, serving as a messenger molecule at lower levels, but can be toxic at higher concentrations.^5^, ^15^ It is known that H_2_O_2_ is generated within a few minutes following herbivory by chewing insects, and its accumulation can trigger the synthesis of phytohormones like jasmonic acid and its derivatives, resulting in the expression of defense‐related genes in local and distal plant tissues that confer induced systemic resistance.^5^, ^16^, ^17^ At elevated concentrations, H_2_O_2_ can react with fatty acids and rupture plant cell membranes, leading to formation of the lipid peroxidation byproduct malondialdehyde (MDA), which is indicative of oxidative stress.^18^, ^19^ Because elevated ROS concentrations are toxic to plant cells, greater activities of antioxidant enzymes can be induced to scavenge excess ROS, highlighting superoxide dismutase (SOD), catalase (CAT), and ascorbate peroxidase (APX).^20^

Several classes of plant defense proteins and secondary metabolites have been reported to play an important role in soybean resistance. Polyphenol oxidases (PPO) are plant defense enzymes that act by oxidizing simple phenolics and converting them into quinones, which are highly reactive compounds correlated with plant resistance to insects.^21^ Proteinase inhibitors are also part of plant defense since these peptides inhibit proteolysis in insect's midgut.^22^ Kunitz trypsin inhibitors (KTI) are serine‐proteinase inhibitors of soybean plants that have antidigestive effects in lepidopteran larvae.^23^, ^24^ Moreover, soybean plants produce a diversity of flavonoids as specialized secondary metabolites.^25^, ^26^ Studies have demonstrated negative effects of soybean flavonoids on the feeding, oviposition, and development of insect species.^27^, ^28^, ^29^ Therefore, the quantification of PPO, KTI, and flavonoids in resistant and susceptible soybean varieties can give insights into their role in constitutive and induced resistance to *S. eridania*.

Previous studies have found soybean lines and commercial varieties with higher levels of constitutive resistance to *S. eridania*.^13^, ^30^ Few studies, however, have evaluated induced resistance in soybean varieties to lepidopteran pest species,^6^, ^7^, ^8^ and none with *S. eridania*. Additionally, there is scarce information in the literature linking plant oxidative stress, defense proteins, and flavonoids with soybean induced resistance to lepidopteran pests.^23^, ^26^, ^31^ This knowledge can aid in the development of protocols for genotype screening and foster the use of soybean varieties with higher induced‐resistance levels to *S. eridania* in integrated pest management.

In this context, given the importance of elucidating the roles of oxidative stress, defense proteins, and flavonoids in soybean induced resistance to *S. eridania*, this study used varieties selected based on prior determinations of differing constitutive antibiosis‐resistance to test the following hypotheses: injury by *S. eridania* larvae induces higher levels of soybean resistance to conspecific larvae; soybean induced resistance is associated with higher activities of the defense proteins SOD, CAT, APX, PPO, and KTI; larval herbivory by *S. eridania* induce higher flavonoid concentrations; and soybean induced resistance trades off with plant growth.

## 2 MATERIALS AND METHODS

### 2.1 Experimental conditions

Experiments were carried out under greenhouse and laboratory conditions in the Department of Plant Protection and Department of Biological Sciences Applied to Agriculture at São Paulo State University, in Jaboticabal, and in the Department of Chemistry of the Federal University of São Carlos, in São Carlos, Brazil. Experiments in the laboratory were conducted in a climate room at 26 ± 2 °C temperature, 70 ± 10% relative humidity, and 12 L:12D h photoperiod, and in the greenhouse under ambient conditions. The experiments with *S. eridania* infestation and evaluation of larval performance were conducted during the summer season, from December 2014 to March 2015.

For all experiments the soybean varieties IAC 100 and BRSGO 8360 were used as resistant and susceptible varieties, based on previous screening trials evaluating constitutive antibiosis‐resistance to *S. eridania*.^13^, ^30^ Four seeds of each soybean variety were sown in 2‐L plastic pots filled with soil, sand, and tanned bovine manure at 3:1:1 ratio. Soil analysis was performed to confirm that fertilization was adequate. Plants were thinned to one plant per pot at the V2 growth stage, i.e., with two expanded trifoliates,^32^ and kept in a greenhouse (2 × 4 × 2 m) covered with anti‐aphid screen. Plants were irrigated on a daily basis, standardizing the same volume to avoid water stress.

Larvae of *S. eridania* used in the experiments were obtained from a colony maintained in the laboratory, for which larvae were initially collected in soybean experimental fields (cv. CD 208) of the São Paulo State University, in Jaboticabal. Larvae were reared on an artificial diet^33^ and supplemented with cotton (cv. FM 339) leaves during the 5th and 6th instars. In the adult stage, *S. eridania* moths were fed on 10% honey solution in soaked cotton wool in plastic cups inside PVC cages (10 × 20 cm). Experiments were initiated using *S. eridania* larvae after three generations of colony acclimation in the laboratory.

### 2.2 Infestation of soybean plants with *S. eridania* larvae in the greenhouse

Soybean plants of both resistant (IAC 100) and susceptible (BRSGO 8360) varieties were assigned to the ‘injured’ and ‘uninjured herbivory’ treatments. Fifty plants were used per herbivory treatment and variety, from which 10 plants were grouped together to make up a repetition, resulting in five true replicates. In total, 200 plants were used in the experiment. These groups of plants were arranged inside the greenhouse in complete randomized design using a 2 × 2 factorial scheme, including the factors ‘variety’ (resistant and susceptible soybeans) and ‘injury’ (injured and uninjured soybeans).

For the soybean plants assigned to the ‘injured’ treatments, plants with three expanded trifoliates (V3 growth stage)^32^ were infested with *S. eridania* larvae. One 5th‐instar larva was transferred onto the central leaflet of the oldest trifoliate of V3‐stage plants and confined in voile‐fabric cages (15 × 20 cm) closed with a twist tie around the petiole. The larvae were removed from the plants after 24 h, sufficient time to inflict ~50% defoliation to the trifoliate. In the ‘uninjured’ plants, cages without larval infestation were used. The youngest expanded trifoliate of soybean plants were collected by excising the petiole with the aid of scissors sterilized with 70% alcohol solution. This trifoliate was collected for use in bioassays and chemical analysis and not the one directly infested by the larvae because the aim was to investigate systemic induced resistance. This procedure of larval infestation was done for all experiments described in the subsequent sections. The infestation methodology was based on previous research demonstrating that soybean plants subjected to larval infestation can express induced resistance in the upper trifoliates of young plants (V3–V4 growth stage).^6^

### 2.3 Bioassay of *S. eridania* larval performance in the laboratory

Three days after injury caused by *S. eridania* 5th‐instar larvae to soybean plants, leaflets from the youngest expanded trifoliate were collected, weighed on analytical scale (Ohaus, Parsipanny, NJ, USA), and placed in Petri dishes (9 cm ø) lined with moist filter paper in the laboratory. The 3‐day post‐injury period was chosen based on previously established findings.^34^ A single neonate larva of *S. eridania* was transferred to a Petri dish, and 50 Petri dishes were prepared per treatment. Each group of 10 Petri dishes made up one true repetition, with five replicates for each treatment. The bioassay was set up in complete randomized design using a 2 × 2 factorial scheme (variety × injury) in a climate room.

Larvae were fed leaflets of the soybean × injury treatments for 5 days, after which the leaf area consumed, leaf dry weight consumed, larval survival, fresh and dry larval weights, and dry frass weight were recorded. Leaf area consumed was measured with an electronic leaf area meter (LI‐COR, Lincoln, NE, USA). For this, the leaf tissues remaining in Petri dishes after larval feeding were scanned and the values subtracted from the total leaf area measured in the beginning of the bioassay. Leaf dry weight consumed, fresh and dry weight of *S. eridania* larvae, and dry frass weight were obtained by weighing the samples on analytical scale (Ohaus, Parsipanny, NJ, USA). For leaf dry weight consumed, dry larval weight, and dry frass weight, samples were dried in an oven at 60 °C for 48 h. Additional 10 plants of each treatment and variety were used as aliquots to estimate leaf dry weight consumed; intact leaves from these plants were collected, dried, and weighed, and then used to calculate the differences from the dry weight of leaf tissues remaining after larval feeding. Larval survival was calculated by subtracting the number of dead larvae from the initial numbers and expressed in percentage.

The values of dry larval weight, dry frass weight, and leaf dry weight consumed were used to calculate the following nutritional indexes: relative consumption rate (RCR); relative metabolic rate (RMR); efficiency of conversion of ingested food (ECI); efficiency of conversion of digested food (ECD); and approximate digestibility.^35^ For the calculations, the initial dry weight of *S. eridania* neonate larvae were considered null.

### 2.4 Quantification of oxidative stress markers and defense proteins

#### 2.4.1 Leaf sample collection

Three days after removing the larvae from soybean plants, the youngest expanded trifoliate was collected with the aid of scissors sterilized with 70% alcohol solution. Leaf collection was performed early in the morning (07:00–09:00) to avoid potential stress caused by higher temperatures. Collected leaf samples were placed in labeled plastic bags and immediately stored in a styrofoam box filled with liquid nitrogen (N_2_) to preserve the metabolic processes. The samples were stored in an ultra‐freezer at −80 °C until analysis.

#### 2.4.2 Extraction and measurements of oxidative stress markers

250 mg of each leaf sample were ground in a mortar under liquid nitrogen (N_2_) until a fine powder was obtained, and then homogenized with 20% polyvinylpolypyrrolidone (PVPP) and 2 mL of 0.1% trichloroacetic acid (TCA). Next, the samples were centrifuged at 12 000 × *g* for 15 min at 4 °C. For H_2_O_2_ measurements, 200 μL of the supernatant were transferred into microcentrifuge tubes containing 200 μL of 100 mM potassium phosphate buffer (pH 7.5) and 800 μL of 1 M potassium iodate, and the samples were incubated on ice in total darkness. For MDA measurements, 250 μL of the supernatant were transferred into microcentrifuge tubes containing 1 mL of solution prepared with 10% TCA and 0.5% thiobarbituric acid (TBA). The samples were homogenized and taken to a water bath at 95 °C for 30 min. The samples were then incubated on ice for 10 min to stop the reaction and centrifuged at 12 000 × *g* for 15 min at 4 °C. The concentrations of H₂O₂^36^ and MDA^37^ were analyzed following established methodologies using three technical replicates for each sample and five biological replicates per treatment.

#### 2.4.3 Extraction and measurements of defense enzymes

Leaf samples were ground in a mortar under N_2_ until a fine powder. Next, potassium phosphate buffer at 100 mM (pH 7.5) containing 1 mM EDTA and 3 mM DTT at 3:1 ratio (buffer: leaf material), plus 4% PVPP were added to the fine powder.^38^ The samples were centrifuged at 10 000 × *g* for 30 min at 4 °C and the supernatant was transferred into 2‐mL microcentrifuge tubes. The activities of the defense enzymes SOD, CAT, APX, and PPO were evaluated using three technical replicates for each sample and five biological replicates per treatment, using a UV‐spectrophotometer (Beckman DU 640, Beckman Instruments Inc., Fullerton, CA, USA). Analyses of the defense enzymes were performed with the following protocols: SOD^39^; CAT^38^; APX^40^; and PPO.^41^ Calculation of the enzyme activities was normalized on the concentrations of total proteins in each treatment following the Bradford method, using bovine serum albumin as standard.^42^

Extraction and measurements of KTI: leaf tissues were ground in a mortar under N_2_ with the addition of 4% PVPP. The obtained fine powder was homogenized using 0.1 M Tris–HCl buffer (pH 8.2) containing 20 mM CaCl_2_ at 3:1 ratio (buffer: leaf material). The samples were centrifuged at 17 200 × *g* for 30 min at 4 °C. The concentrations of KTI were determined using two technical replicates for each sample and five biological replicates per treatment, using bovine trypsin (Sigma Aldrich Saint Louis, MO, USA) as standard, following previously published protocols.^23^, ^43^ The activities of KTI were calculated based on the following equation: (A × B) / (C × 1000 × P); where A = absorbance value in the control (blank) minus the absorbance value in the treatments (soybean plants); B = dilution used; C = trypsin factor (0.019, which is the product of the action of 1 ug trypsin on BApNA that results in 0.019 absorbance units at 410 nm); and P = protein concentration (g mL^−1^).^23^

### 2.5 Quantification of flavonoids

The flavonoids rutin, daidzin, hesperidin, and isoquercitrin were quantified by high‐resolution mass spectrometry. These flavonoids were selected based on their contrasting concentrations previously found in the soybean varieties IAC 100 and BRSGO 8360 and the potential role in plant resistance to lepidopteran larvae.^25^, ^29^

Soybean plants were grown in the greenhouse as previously described. Three days after removing *S. eridania* larvae from the plants, the youngest expanded trifoliate was collected using scissors sterilized with 70% alcohol solution, stored in labeled plastic bags, and immediately taken to the laboratory. Each true replicate was composed of seven trifoliates collected from seven potted plants, and five replicates were used per treatment. Leaf samples were dried to constant weight in an oven at 40 °C for 72 h, and ground to a fine powder with the aid of a knife mill (model Te650, Thomas Wiley Mill, Swedesboro, NJ, US). The samples were transferred to labeled plastic bags, wrapped in aluminum foil, and stored in a laboratory room at ambient temperature without light exposure.

Extraction of flavonoids was done in two steps. Initially, 400 mg of each sample was weighed, transferred to 15‐mL Falcon tubes, and mixed with 8 mL of 0.5 M MeOH/HCl 80:20 (v/v) solution. The samples were homogenized in Ultra‐Turrax (T‐10 Basic Ultra‐Turrax, IKA, China) for 2 min, and then centrifuged at 10 000 *g* for 30 min at 18 °C. The supernatant was separated and stored in Falcon tubes. The resultant precipitate was subjected to another extraction with 8 mL 0.5 M MeOH/HCl 95:5 (v/v) solution. The samples were homogenized in Ultra‐Turrax for 30 s, kept at rest for 30 min, and centrifuged at 10 000 × *g* for 30 min at 18 °C. The precipitate resultant from this extraction process was discarded, and the supernatant was mixed with the supernatant obtained from the first extraction, amounting 13 mL volume. The total volume of each sample was divided in three 4‐mL HPLC flasks and dried overnight (~15 h) in SpeedVac. Sedimented pellets were suspended in a mixture of 500 μL of MeOH and 500 μL distilled water. They were filtered through a 0.2‐μm sieve, dried again, and stored until their analysis.

The selected flavonoids were analyzed by ultra‐performance liquid chromatography (Waters Acquity, Water Corp., Millford, MA, USA) coupled with high‐resolution mass detector (Waters Xevo G2‐SX QTOF, Waters Acquity, Waters Corp., Millford, MA, USA), with an electrospray ionization analyzer in both positive and negative modes. The dried samples were weighted, resuspended in MS‐grade H_2_O/MeOH (9:1) solution, and adjusted to a final concentration of 1.0 mg mL^−1^. Chromatographic optimization was performed in exploratory gradient using the column Acquity UPLC BEH BEH C_18_ (130 Å, 1.7 μm, 2.1 mm × 50 mm, Waters, Waters Corp., Millford, MA, USA). We used 20 μL of injection volume, and the eluents used for separation were ultrapure water Milli‐Q (A) and acetonitrile (B), both acidified with 0.1% formic acid.

The interval chosen for data acquisition in the mass detector was 100–1200 Da, alternating the energy of scans from high to low (MSE mode). The low collision energy was set to 6 eV, while the high voltage energy was 25 eV. Scan time for each function was 10 s. Leucine enkephalin m/z 556.2766 (200 pg μl^−1^) was used as standard for Lock mass. The source temperature was 100 °C and the solvation temperature 450 °C, with 750 L ha^−1^ of N_2_ flux. The capillary and cone voltages were 2.5 kV and 30 V, respectively. Data acquisition was controlled by UNIFI 1.8 platform. A relative quantitative analysis of the selected flavonoids was performed by integrating the chromatographic peak areas obtained by mass spectrometry and comparing their signal intensities across the different treatments.

### 2.6 Evaluation of soybean plant growth

Plant growth parameters were recorded in resistant and susceptible soybean plants injured or not by *S. eridania* larvae to confirm if resistance induction negatively affects plant growth. For this, 60 plants of both varieties were grown in the greenhouse as previously described, and were assigned to the ‘injured’ and ‘uninjured’ treatments. Thirty plants were used for each variety × injury treatment combination, from which six plants were grouped together to make up a repetition, totaling five true replicates in complete randomized design and 2 × 2 factorial scheme.

The growth parameters plant height (cm), time to flowering (days), total leaf area (cm^2^), and leaf dry weight (g) were recorded in the soybean plants when they reached the R1 flowering stage.^32^ Plant height was measured with a ruler from the base close to soil until the apical meristem. Total leaf area was evaluated with the aid of an electronic leaf area meter (LI‐COR, Lincoln, NE, USA) after removing all leaves from the plants. The collected leaves were dried in an oven at 60 °C for 48 h and weighed on analytical scale. The oldest trifoliate of soybeans that were directly infested by *S. eridania* (injured plants) or that received only the cages (uninjured plants) were not included in the evaluations of total leaf area and leaf dry weight.

### 2.7 Statistical analysis

Data obtained in the experiments were checked for residual distribution and homogeneity of variances using the Kolmogorov–Smirnov and Levene tests, respectively. Data were analyzed by two‐way ANOVA to account for the main effects of soybean variety, injury, and the variety × injury interactive effects. Means of treatments were compared by Tukey HSD test (*α* = 0.05) when ANOVA was significant. Data of chemical analysis were also analyzed by principal component analysis (PCA), separating the treatments according to their similarity. Statistical analysis was performed in Statistica version 7.0.^44^

## 3 RESULTS

### 3.1 Bioassay of *S. eridania* larval performance in the laboratory

**Figure 1:**
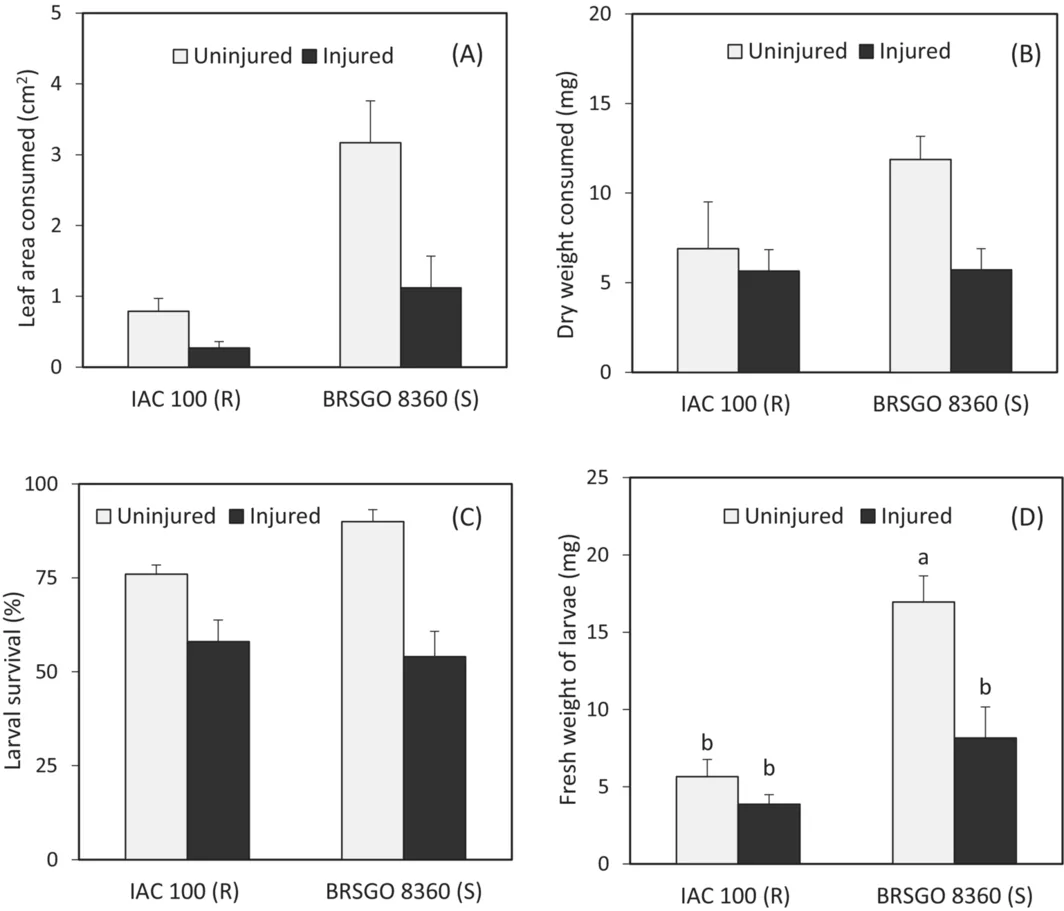
Leaf area consumed (A), dry leaf weight consumed (B), larval survival (C), and fresh larval weight (D) of *S. eridania* fed leaves of resistant and susceptible soybean varieties injured or not by *S. eridania* larvae. Bars topped with different lowercase letters differ by Tukey test

Leaf area consumed by *S. eridania* larvae was affected by soybean variety and by previous injury caused by conspecific larvae, and these effects were independent (Table 1). Leaves of the resistant variety IAC 100 had a smaller area consumed; likewise, in both varieties, previously injured plants showed lower leaf area consumed (Fig. 1(A)). In contrast, consumed leaf dry weight was not affected by either soybean variety or previous injury (Table 1, Fig. 1(B)).

**Table 1:** Two‐way ANOVA values for the effects of variety (V), injury (I), and variety × injury (V × I) interaction for biological parameters and nutritional indexes of *S. eridania*, and soybean defense proteins, oxidative stress markers, flavonoids, and plant growth parameters

Sources of variation	df	Leaf area consumed		Leaf weight consumed		Larval survival		Larval weight	
		*F*	*P*	*F*	*P*	*F*	*P*	*F*	*P*
V	1	24.05	**0.0002**	2.68	0.1208	1.04	0.3226	28.63	**<0.0001**
I	1	17.63	**0.0007**	3.34	0.0662	30.38	**<0.0001**	13.17	**0.0023**
V × I	1	0	0.9923	2.35	0.1449	3.38	0.0848	5.8	**0.0284**

Sources of variation	df	RCR		RMR		ECI		ECD	
		*F*	*P*	*F*	*P*	*F*	*P*	*F*	*P*
V	1	0.29	0.5962	2.30	0.1517	6.85	**0.0186**	1.21	0.2895
I	1	1.55	0.2306	0.82	0.3791	1.55	0.2315	3.15	0.0978
V × I	1	0.12	0.7323	0.01	0.9347	0.03	0.8588	0.01	0.9419

Sources of variation	df	AD		H_2_O_2_		MDA		SOD	
		*F*	*P*	*F*	*P*	*F*	*P*	*F*	*P*
V	1	15.93	**0.0013**	0.22	0.6444	91.17	**<0.0001**	22.3	**0.0002**
I	1	18.52	**0.0007**	5.47	**0.0327**	16.52	**0.0009**	6.32	**0.0230**
V × I	1	3.11	0.0998	1.04	0.3241	0.17	0.6830	0.17	0.6815

Sources of variation	df	CAT		APX		PPO		KTI	
		*F*	*P*			*F*	*P*	*F*	*P*
V	1	20.41	**0.0004**	62.88	**<0.0001**	72.17	**<0.0001**	32.02	**<0.0001**
I	1	2.05	0.1714	2.01	0.1751	4.22	**0.0566**	0.44	0.5164
V × I	1	0.59	0.4550	0.59	0.4548	0	0.9642	4.04	**0.0519**

Sources of variation	df	Rutin		Hesperedin		Daidzin		Isoquercetrin	
		*F*	*P*	*F*	*P*	*t*	*P*	*t*	*P*
V	1	0.13	0.7209	0.89	0.3592	‐	‐	‐	‐
I	1	0.04	0.8368	1.05	0.3204	−1.80	0.1095	1.29	0.2324
V × I	1	1.85	0.1925	3.45	0.0819	‐	‐	‐	‐

Sources of variation	df	Plant height		Days to flowering		Total leaf area		Plant dry weight	
		*F*	*P*	*F*	*P*	*F*	*P*	*F*	*P*
V	1	12.35	**0.0029**	57.94	**<0.0001**	27.94	**<0.0001**	10.91	**0.0045**
I	1	0.08	0.7786	0.04	0.8352	0	0.9790	0.20	0.6623
V × I	1	0.87	0.3647	0.19	0.6701	0.05	0.8235	0	0.9734

Values in bold indicate significant differences (*P* < 0.05) by ANOVA.

Larval survival was significantly affected only by injury (Table 1). Larvae of *S. eridania* that fed uninjured plants of the resistant and susceptible varieties showed survival rates 1.3‐ and 1.7‐fold greater than the survival in injured plants of the respective varieties (Fig. 1(C)). The effects of soybean variety and variety × injury interaction were not significant for larval survival.

Larval fresh weight of *S. eridania* significantly differed between soybean varieties and between injured and uninjured plants. The effects of variety and injury were dependent of each other (Table 1). In the resistant variety, there was no difference in larval fresh weights between injury conditions, whereas in the susceptible variety the larvae showed weight gain 2.1‐fold greater in uninjured as compared to injured soybeans (Fig. 1(D)). Soybean variety affected the performance of *S. eridania* in terms of leaf consumption and larval weight, with no effect on larval survival. In contrast, previous injury by *S. eridania* also negatively affected survival.

Soybean variety affected the efficiency of conversion of ingested food (ECI) and both variety and injury affected approximate digestibility (AD) in *S. eridania* larvae. The effects were independent for the latter parameter (Table 1). The other nutritional indexes assessed, namely relative consumption rate (RCR), relative metabolic rate (RMR), and efficiency of conversion of digested food (ECD) were not significantly influenced by soybean variety and injury (Table 1).

Larvae of *S. eridania* fed leaves of the susceptible variety showed ECI values nearly 4‐fold greater than those fed leaves of the resistant variety (Table 2). Larvae that fed on the susceptible variety had AD values >2‐fold greater than those fed the resistant variety (Table 2). Also, the AD values were 2.3‐fold greater in *S. eridania* larvae fed leaves of uninjured plants relative to larvae fed injured ones (Table 2). The results of the nutritional indexes correlated with the negative effects of the resistant variety on larval performance, as well as with the negative effects of previous injury. These relationships may be driven by the lower digestibility of leaf tissues in these treatments, resulting in reduced efficiency of larvae in converting ingested foliage into biomass.

**Table 2:** Nutritional indexes of *S. eridania* neonate larvae fed leaves of resistant (R) and susceptible (S) soybean varieties previously injured or not by conspecific larvae

Nutritional indexes	IAC 100 (R)		BRSGO 8360 (S)	
	Injured	Uninjured	Injured	Uninjured
RCR (mg mg^−1^ day^−1^)	6.08 ± 2.88	4.43 ± 2.02	7.88 ± 4.15	2.12 ± 0.52
RMR (mg mg^−1^ day^−1^)	0.04 ± 1.40	1.16 ± 1.20	1.77 ± 1.67	0.77 ± 0.25
ECI (%)^a^	6.87 ± 2.68	9.40 ± 2.09	25.62 ± 9.78	38.92 ± 19.82
ECD (%)	−11.70 ± 17.07	9.82 ± 10.34	−63.55 ± 65.76	26.51 ± 5.83
AD (%)^b^	3.03 ± 7.87	48.73 ± 10.59	46.38 ± 4.45	65.53 ± 6.78

^a^ Significant difference (*P* < 0.05) for variety.

^b^ Significant differences (*P* < 0.05) for variety and injury.

### 3.2 Quantification of oxidative stress markers and defense proteins

**Figure 2:**
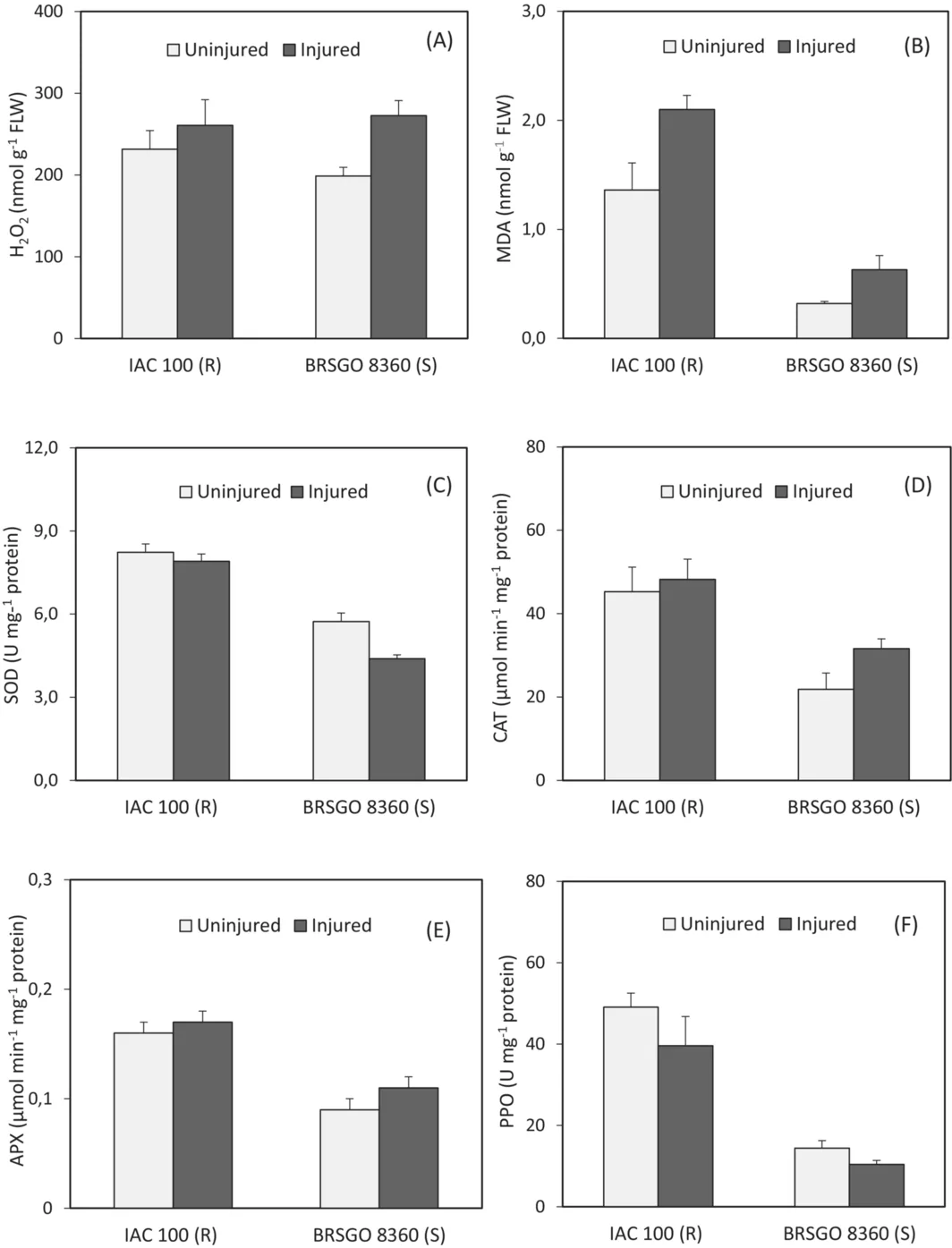
Concentrations of H_2_O_2_ (A) and malondialdehyde (MDA) (B), and activities of the enzymes superoxide dismutase (SOD) (C), catalase (CAT) (D), ascorbate peroxidase (APX) (E), and Polyphenol oxidases (PPO) (F) in soybean plants of resistant and susceptible varieties injured or not by *S. eridania* larvae.

The concentrations of H_2_O_2_ and MDA were significantly affected by *S. eridania* herbivory on soybean plants (Table 1). Injured plants showed H_2_O_2_ and MDA concentrations on average 19.2% (Fig. 2(A)) and 42.2% (Fig. 2(B)) greater relative to uninjured plants, respectively. MDA concentrations were constitutively superior also in the resistant variety. There were no significant interactive effects of variety and injury for the concentrations of H_2_O_2_ and MDA (Table 1). These results indicated that H_2_O_2_ concentrations were enhanced following herbivory, and likely contributed to the signaling of induced resistance expression. Other processes, independently of H_2_O_2_ oxidative damage, may also have affected MDA concentrations such that great constitutive levels were observed in the uninjured resistant variety.

All tested enzyme activities were significantly higher in the resistant soybean variety than in the susceptible variety (Table 1, Fig. 2). Only superoxide dismutase (SOD) activity was also influenced by previous *S. eridania* injury (Fig. 2), being 17.2% higher in uninjured plants than in injured ones. Similarly, polyphenol oxidase (PPO) activity was affected by previous injury, although only marginally (*P* = 0.0566) (Fig. 2). There were no interactive effects of soybean variety and injury for SOD, CAT, APX, and PPO (Table 1). According to the obtained data, the defense enzyme activities can explain the higher constitutive resistance levels in the soybean varieties, but did not correlate with induced resistance expression.

**Figure 3:**
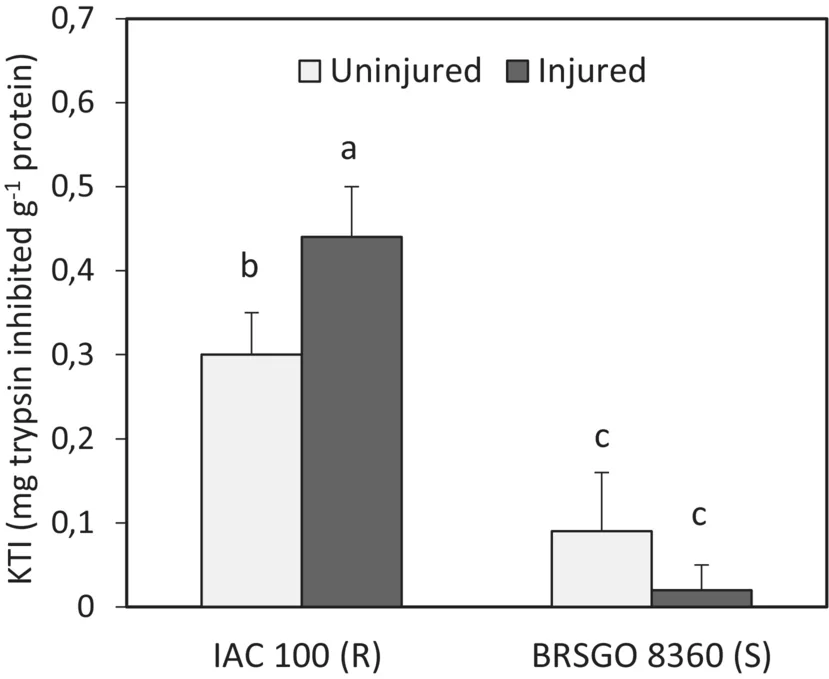
Activities of Kunitz trypsin inhibitors (KTI) in plants of soybean resistant and susceptible varieties injured or not by *S. eridania* larvae. Bars topped with different lowercase letters differ by Tukey test.

KTI activities significantly differed between soybean varieties (Table 1). The activity in the resistant variety IAC 100 was 6.7‐fold higher than in the susceptible variety BRSGO 8360 (Fig. 3). In addition, there was a marginally significant difference (*P* = 0.0519) for the variety × injury interaction (Table 1). Injured plants of the resistant variety showed increased KTI activities, whereas in the susceptible soybean no differences were found between injury conditions (Fig. 3). Therefore, there is evidence that greater KTI activities in injured plants of the resistant soybean variety correlate with the lower digestibility of leaf tissues, the lower efficiency of conversion of ingested food into larval weight, and the overall negative impact on *S. eridania* larval performance.

### 3.3 Quantification of flavonoids

The results for the chromatograms and retention times of the flavonoids rutin, daidzin, hesperidin, and isoquercitrin in plants of soybean varieties injured or not by *S. eridania* larvae are presented in Figs S1–S8. All soybean variety × injury treatments presented the flavonoids rutin and hesperidin; in contrast, isoquercitrin was only identified in the resistant variety and daidzin in the susceptible variety, under both injury conditions.

There were no significant differences in variety, injury, and variety × injury for rutin. Similarly, hesperidin concentrations were not affected by these factors and their interaction. The flavonoids daidzin and isoquercitrin were only analyzed for the effect of injury in each of the variety they were detected. Larval herbivory did not affect isoquercitrin and daidzin concentrations in the resistant and susceptible varieties, respectively (Table 1). Among the selected flavonoids, only isoquercitrin was found to be a good proxy of soybean constitutive resistance to *S. eridania*, and none of them was associated with induced resistance.

**Figure 4:**
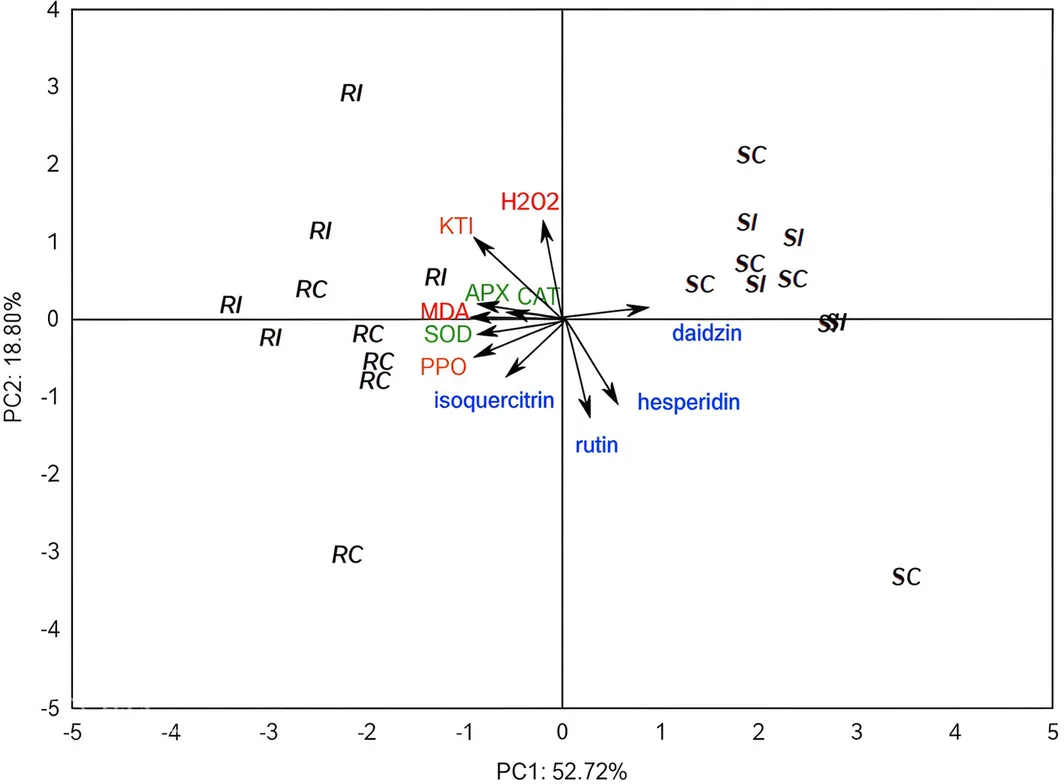
Principal components analysis of plant stress markers, defense proteins, and flavonoids in resistant and susceptible soybean varieties injured or not by *S. eridania* larvae. RI, injured resistant variety; RC, control resistant variety; SI, injured susceptible variety; SC, control susceptible variety.

The PC1 and PC2 components together explained 71.52% data variation of the evaluated chemical compounds. PC1 mostly accounted for data variability between soybean varieties, while PC2 better contrasted the effects of larval injury in treatment distribution among quadrants. Data from the resistant variety were separated in the second and third quadrants and data of the susceptible variety were mostly grouped in the first quadrant (Fig. 4).

The distribution of the variety × injury treatment combinations generated by the PCA summarized the main results of the univariate analyses. The activities of the defense proteins SOD, CAT, APX, PPO, and KTI, and MDA concentrations were the most influential parameters in separating the soybean varieties along the PC1 axis, directly correlating with higher amounts in the resistant variety. In addition, KTI was associated with injured resistant soybeans. H_2_O_2_ concentrations most affected treatment distribution along the PC2 axis, such that injured soybeans of both varieties possessed higher levels. The flavonoids isoquercitrin and daidzin were exclusive to the resistant and susceptible varieties, respectively, being their distribution inversely correlated. Rutin and hesperidin concentrations were associated with uninjured soybean plants, mainly in the susceptible variety (Fig. 4).

### 3.4 Evaluation of soybean plant growth

**Figure 5:**
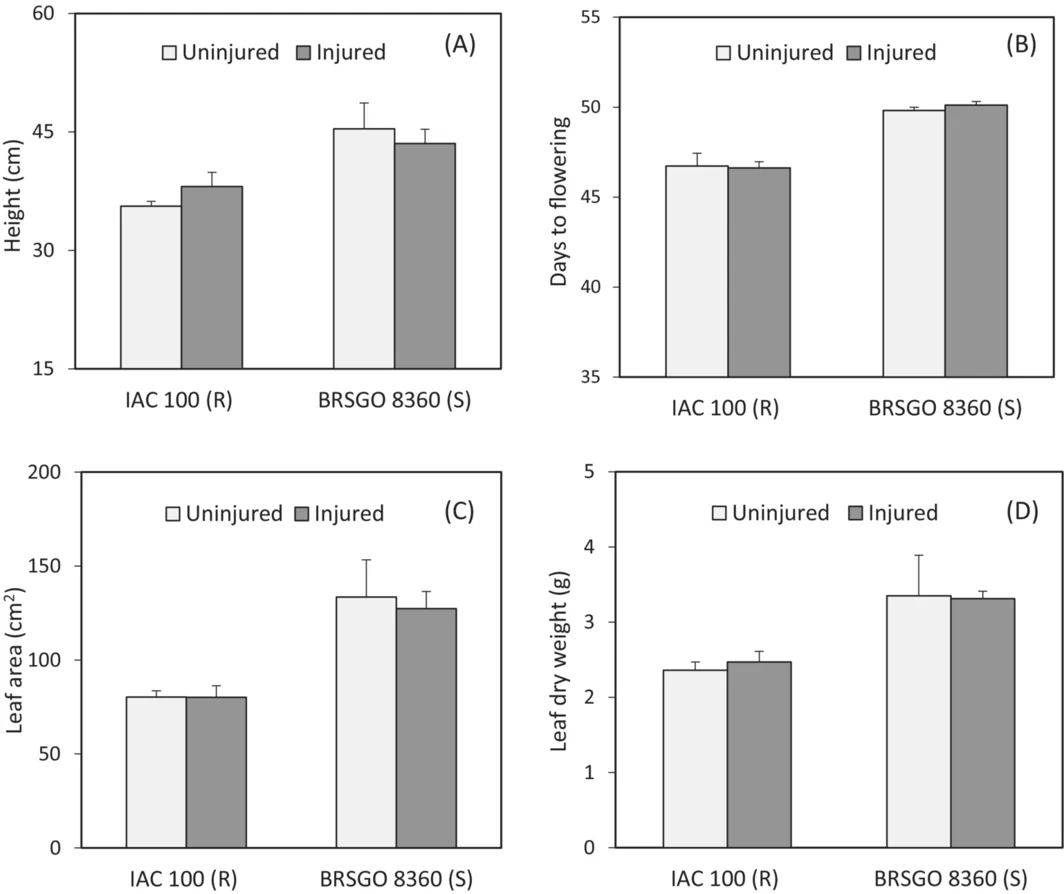
Plant height (A), days to flowering (B), total leaf area (C), and leaf dry weight (D) of plants of resistant and susceptible soybean varieties injured or not by *S. eridania* larvae.

Plant growth parameters were recorded in the soybean variety × injury treatment combinations with the aim of assessing if resistance induced by *S. eridania* herbivory was associated with lower plant growth. Larval injury did not significantly affect any of the plant growth parameters. On the other hand, there were significant variety effects for plant height, time to flowering, plant dry weight, and total leaf area. There were no significant soybean variety × injury interactive effects (Table 1). The mean values of plant height (Fig. 5(A)), days to flowering (Fig. 5(B)), total leaf area (Fig. 5(C)), and leaf dry weight were all superior in the susceptible soybean variety.

## 4 DISCUSSION

Induced resistance expressed in soybean plants after *S. eridania* herbivory negatively affected the performance of conspecific larvae. In addition, there was a great difference in larval performance between the soybean varieties IAC 100 and BRSGO 8360, confirming their respective constitutive resistance and susceptibility levels. Larvae of *S. eridania* that fed leaves from the susceptible variety or from uninjured soybeans showed greater leaf consumption and weight gain, whereas larval survival was only affected by prior herbivory. These effects coincided with higher approximate digestibility in the susceptible variety and uninjured soybeans. Moreover, larvae fed the susceptible variety exhibited 4‐fold higher efficiency in converting ingested leaf tissue to biomass than the resistant variety. These results suggest that digestibility of leaf tissues and nutrient assimilation by *S. eridania* larvae were impaired in the variety IAC 100 and injured soybeans, and these negative effects resulted from the expression of constitutive and induced resistance, respectively. It is important to highlight that induced resistance was systemically manifested in the soybean plants, since leaflets used in the bioassays were not directly infested by the larvae.

Soybean plants have been investigated for induced resistance to different herbivorous insects, including leaf beetles,^34^ lepidopteran larvae,^6^, ^7^, ^8^, ^23^, ^45^, ^46^ aphids,^47^ stink bugs,^48^ and thrips.^26^ Despite the number of studies and target pests, a minority of studies have evaluated induced resistance in soybean varieties differing in constitutive resistance levels and established the concerted role of oxidative stress markers, defense proteins, and flavonoids as the underlying chemical mechanisms.^23^, ^48^, ^49^ Although all treatments in our study followed the same standardized method of leaf collection, we acknowledge that we did not include a treatment with mechanical injury to isolate the effects of general wounding from those of insect herbivory. Therefore, we cannot determine whether the effects on larval performance observed in previously injured plants were due to mechanical damage caused by leaf removal or to defenses activated by factors specific to larval feeding, such as elicitors in oral secretions.

Distinct plant genotypes can show varying induced‐resistance levels to herbivorous insects, since the outcome depends on the differential expression and allelic variation of defense‐related genes present in their genomes.^50^ Although it is suggested that there are fitness costs between constitutive and induced resistance, this issue is often debated given the contrasting results found in the literature.^2^, ^6^, ^8^, ^9^ In our study, both soybean varieties showed increased resistance levels after *S. eridania* herbivory, and the constitutive‐resistant variety showed greater inducibility. A study evaluating 18 soybean genotypes using 2‐mM jasmonic acid solution applied to the plants reported that most genotypes successfully induced resistance to *Spodoptera frugiperda* (J.E. Smith), with the exception of Asgrow 5533.^8^ In another study with distinct soybean varieties, the constitutive‐resistant line PI 227687 showed induced resistance following *Spodoptera cosmioides* (Walker) herbivory, whereas the susceptible variety P98Y11RR did not display this trait. This study also reported that resistance was induced only in young leaves when larvae defoliated older leaves of V3–V4 growth‐stage plants, but not the inverse.^6^ Therefore, in addition to the temporal basis of induced resistance, soybeans also show systemic variability related to leaf ontogeny.^34^

Greater concentrations of H_2_O_2_ and MDA were found in the injured compared to uninjured soybeans, indicating that *S. eridania* larval herbivory caused plant stress, leading to systemic accumulation of reactive oxygen species, and ultimately oxidative damage in the leaf tissues. In the injured plants, H_2_O_2_ probably played an important role as a signaling molecule in the early steps of injury perception, being responsible for signal transduction and expression of resistance genes by the coordinated action of phytohormones.^5^, ^51^ We hypothesize that elevated H_2_O_2_ concentrations in leaf tissues of the injured plants may have directly affected *S. eridania* larval performance. Previous studies reported that H_2_O_2_ has antimicrobial properties and that the oxidative damage caused in plant tissues can impair insect cells,^5^, ^31^, ^52^ but this hypothesis warrants further investigation.

Reactive oxygen species (ROS) are naturally produced at low concentrations in plant cells as byproducts of aerobic metabolism and photosynthesis.^53^ ROS concentrations can increase considerably upon stress, leading to oxidative damage due to lipid peroxidation, protein oxidation, and cellular apoptosis. Despite being toxic to the plants at high concentrations,^5^, ^31^ low ROS levels act as signaling molecules, activating plant physiological processes and induced systemic resistance in localized and distal plant tissues.^54^, ^55^ Malondialdehyde (MDA) is one of the final peroxidation products of polyunsaturated fatty acids, being synthesized when the ROS levels surpass the concentrations that plants can scavenge through antioxidant metabolism.^18^ Quantification of H_2_O_2_ and MDA concentrations has been used as proxy of plant oxidative stress and induced defense responses to insect herbivory in many studies.^19^, ^56^, ^57^

Interestingly, MDA contents were constitutively high in the resistant variety, and further increased with larval herbivory. It is tempting to speculate that higher MDA concentrations are associated with increased resistance levels in the variety IAC 100. MDA is a toxic compound generated during lipid peroxidation of membranes of plant cells and organelles caused by ROS damage.^19^ We hypothesize that greater MDA levels are produced in the resistant soybean variety because of greater synthesis of jasmonic acid (JA), the major phytohormone involved in plant resistance to insects.^58^ JA is an oxylipin synthesized through the octadecanoid pathway that is derived from the fatty acid linolenic acid present in the membranes.^59^ Previous research demonstrated that constitutive concentrations of JA and H_2_O_2_ were greater in the maize genotype Mp708 resistant to *S. frugiperda* than in the susceptible inbred line Tx601, suggesting these chemical mechanisms can play a role in priming insect‐resistance responses.^60^, ^61^ Thus, we hypothesize that the greater constitutive MDA concentrations in the resistant soybean variety IAC 100 may have been due to prior H_2_O_2_ peroxidation or by the action of lipoxygenases and phospholipases, resulting in greater JA production and constitutive resistance.^53^, ^59^ Further studies are needed to investigate if MDA concentrations can exert direct toxicity to insects given the limited information in the literature.

The antioxidant enzyme activities of CAT and APX did not increase in soybeans after *S. eridania* herbivory. By contrast, SOD activities decreased in the injured plants. The activities of SOD, CAT, and APX were constitutively higher in the resistant variety, which also showed greater MDA concentrations than the susceptible variety, but the varieties did not differ in H_2_O_2_ contents. The lower SOD activities in the injured plants may have been due to competition between antioxidant enzymes, such that others may have been upregulated to act in ROS removal.^62^ Alternatively, SOD may have been expressed at higher levels right after *S. eridania* initial herbivory to the plants, but were not detected due to the time period assessed. SOD is the first antioxidant enzyme activated after plant stress to convert O_2_^−^ into O_2_ and H_2_O_2_,^63^ and was found to be triggered by insect herbivory.^64^ This may be the case of CAT and APX as well. Because we only recorded the enzyme activities at one time point, i.e., 3 days after injury, it is possible that during this time interval the activities could have been increased earlier after herbivory and then decreased. Another possible hypothesis is that the activities of antioxidant enzymes were not activated right after herbivory to promote H_2_O_2_ and MDA accumulation, and consequently these greater concentrations were perceived as stress signals for soybean plants to induce resistance.

The resistant soybean variety IAC 100 and the susceptible variety BRS Silvania RR did not show significant changes in APX activity following injury by *Euschistus heros* (Fabricius).^48^ The activities of CAT were similar between injured and uninjured plants of the resistant variety, whereas in the susceptible variety *E. heros* injury reduced the enzyme activity.^48^ On the other hand, soybean plants exhibited an increase in APX activity and a decrease in CAT activity after larval injury by *Helicoverpa zea* (Boddie).^31^ Thus, both cited results are compatible with those of our study. Evaluating the activities of these and other defense enzymes across different time points after insect injury can yield more conclusive results about their participation in soybean induced resistance.

The activities of polyphenol oxidase (PPO) and Kunitz trypsin inhibitor (KTI) were measured in soybean plants as they participate in plant resistance to insects. PPO and KTI activities were significantly greater in the resistant soybean variety, which can explain its constitutive resistance to *S. eridania*. Regarding their roles in induced resistance, contrasting outcomes were found; marginally significant effects were observed for injury on PPO and for variety × injury on KTI. The activities of PPO were reduced after larval herbivory in both soybean varieties. On the other hand, KTI activity increased with *S. eridania* herbivory only in the resistant variety.

PPO acts by oxidizing simple phenolics and converting them into quinones, which are known to be toxic molecules. In addition, PPO can alkylate proteins, decreasing the nutritional quality of ingested food by limiting amino acid availability to insects.^21^ In the current study, although PPO levels can underly soybean constitutive resistance, the enzyme activity was reduced after *S. eridania* herbivory. Similar results were observed following *H. zea* larval injury in soybean plants,^31^ with no significant changes in PPO activity. KTI is a major serine‐proteinase inhibitor found in leaf tissues, reproductive organs, and storage structures of soybean plants, impairing trypsin activity in lepidopteran larvae's midgut. KTI activities were increased in two soybean varieties after *S. frugiperda* herbivory.^26^ Thus, KTI increases following larval herbivory in our study can help explain the greater negative effects of injured resistant soybeans on *S. eridania* performance.

The presence of the flavonoids isoquercitrin and daidzin differed between soybean varieties. Isoquercitrin was only detected in the resistant variety IAC 100, whereas daidzin was found only in the susceptible variety BRSGO 8360. Both flavonoids were present in the injured and uninjured plants of these varieties. By contrast, rutin and hesperidin concentrations were not significantly influenced by soybean variety, larval injury, or their interaction.

The flavonoid daidzin was not detected in intact plants of the resistant variety IAC 100; however, plants injured by *Nezara viridula* (Linnaeus) exhibited highly induced daidzin concentrations, suggesting that this resistance mechanism in soybean is activated only after stink bug infestation.^27^ Rutin concentrations were reduced in plants of seven soybean genotypes infested by *Bemisia tabaci* (Gennadius) compared with uninfested plants. Depending on the genotype, rutin levels varied substantially across plant growth stages, and it was concluded that the relationship between flavonoids and whitefly‐resistance is not yet fully established.^28^ Although the evaluated flavonoids were previously associated with soybean resistance,^27^, ^29^, ^65^ it is possible that their effects are variety‐specific or dose‐dependent, making them stimulants or detrimental in function of the concentrations in plant tissues.^66^

The growth of soybean plants injured at an early stage (~50% defoliation of the oldest trifoliate of V3‐stage plants) by *S. eridania* larvae was not affected by herbivory. All evaluated plant growth parameters, namely plant height, time to flowering, total leaf area, and leaf dry weight, differed between varieties, with susceptible plants showing superior growth. These results demonstrate that the susceptible soybean plants have faster intrinsic rates of growth and development, suggesting a trade‐off with the expression of constitutive resistance.

One of the main assumptions in plant‐insect interactions is that expression of resistance traits poses fitness costs to plant development, such that plants that do not continuously invest resources into chemical and morphological defenses against herbivorous insects are favored by natural selection in the absence of herbivory.^9^ Because plants make use of the same resources (e.g., water, inorganic nutrients, and organic photoassimilates) to invest in defense and growth processes, it is expected that expression of constitutive and induced resistance is energetically costly, reducing plant growth, seed production, and germination.^10^, ^45^ The deployment of induced resistance mechanisms is considered an adaptive defense strategy, in which uninfested plants can save energy and resources to allocate to growth when the risk of herbivory and damage is low.^11^ However, this hypothesis was only partially investigated in our study since we did not measure the seed production and germination in infested and uninfested soybean plants. Moreover, we just compared two varieties to draw consistent conclusions on the evolutionary cost of resistance on plant fitness.

In our study, both soybean varieties induced resistance to *S. eridania*, with greater impacts caused by the resistant variety on larval performance. Therefore, the constitutively resistant variety also showed higher induced resistance levels. Conversely, the resistant variety showed intrinsically lower plant growth than the susceptible variety, which suggests a trade‐off with constitutive resistance. Soybean plants of both varieties injured by *S. eridania* did not experience significant impairment of growth and development. Similar results were obtained when evaluating the field application of resistance elicitors on soybeans and their impacts on *Chrysodeixis includens* (Walker) larval performance, with no negative effects of induced resistance expression on grain yield.^46^ Therefore, according to the experimental conditions used herein, soybean induced resistance to *S. eridania* larval herbivory did not trade‐off with plant growth, contrary to our expectations.

Important caveats should be made. First, the soybean plants were infested with *S. eridania* larvae only once during the experiment. Although the percentage of defoliation was considerable (i.e., ~50% of the trifoliate, and ~ 25% of the whole plant) and near the economic thresholds for lepidopteran larvae species,^14^, ^67^ in field conditions, the plants can suffer additional insect infestation, which can further compromise plant growth. Second, we excluded from the evaluation the trifoliate directly injured by the larvae to standardize with the uninfested plants. Third, varying defense responses can occur across plant development, such that the growth stage, age of leaf tissue, and type of plant structure can influence induced resistance and compensatory growth.^68^ Finally, we cannot rule out the potential costs and effects of tolerance, whose physiological mechanisms can be activated after herbivory,^69^ and are often neglected due to the difficulty of quantification.^9^

Based on the results of plant growth, we hypothesize that the antioxidant enzymes may have been upregulated in later stages after herbivory, which contributed to remove excess ROS and resume normal plant development. Previous studies have documented the role of antioxidant enzymes, especially peroxidases, in soybean tolerance to aphids.^70^, ^71^ Therefore, future research should focus on the evaluation of antioxidant enzyme activities across different time points after herbivory and their correlation with compensatory plant growth to investigate if they play a role in soybean tolerance to insect attack.

In summary, the concentrations of H_2_O_2_ were enhanced in soybeans after *S. eridania* herbivory, which may have been involved in the early signaling of resistance induction. MDA concentrations were constitutively higher in the resistant variety and increased in plants injured by *S. eridania*. The defense proteins SOD, CAT, APX, PPO, and KTI play an important role in soybean constitutive resistance. Only the activities of PPO and KTI were altered with larval herbivory, whereby PPO activities overall reduced, and KTI only increased in the resistant variety. The flavonoid isoquercitrin was associated with constitutive resistance. There was no evidence of plant growth trade‐off with induced resistance after *S. eridania* herbivory.

The findings of this study can aid in the development of protocols for high‐throughput phenotyping in soybean breeding programs by using the highlighted chemical compounds as reliable markers of constitutive and induced resistance. These chemical markers could also be used for confirming resistance induction with the application of defense elicitors, such as jasmonic acid and others, fostering the use of induced resistance in soybean integrated pest management.

## AUTHOR CONTRIBUTIONS

BHSS conceptualized the study, conducted research, analyzed data, wrote and edited the manuscript. ALBJ conceptualized the study and reviewed the manuscript. DMMS analyzed enzyme activities. MRF analyzed flavonoids. APN analyzed oxidative markers and enzyme activities. AFL reviewed and edited the manuscript. MJS conceptualized the study and reviewed the manuscript.

## FUNDING INFORMATION

The study was funded by the São Paulo Research Foundation (Fundação de Amparo à Pesquisa de São Paulo – FAPESP, Process no. 14/11700‐6), the Coordination for the Improvement of Higher Education Personnel (Coordenação de Aperfeiçoamento de Pessoal de Nível Superior‐CAPES), and the National Council for Scientific and Technological Development (Conselho Nacional de Desenvolvimento Científico e Tecnológico‐CNPq).

## CONFLICT OF INTEREST

The authors have no conflict of interest to declare.

## Supporting information


**Figure S1.** Chromatogram of ions extraction of the flavonoids hesperidin (1) and rutin (2) in leaf sample replicates of injured resistant soybean.
**Figure S2.** Chromatogram of ions extraction of the flavonoid isoquercitrin in leaf sample replicates of injured resistant soybean.
**Figure S3.** Chromatogram of ions extraction of the flavonoids hesperidin (1) and rutin (2) in leaf sample replicates of uninjured resistant soybean.
**Figure S4.** Chromatogram of ions extraction of the flavonoid isoquercitrin in leaf sample replicates of uninjured resistant soybean.
**Figure S5.** Chromatogram of ions extraction of the flavonoids hesperidin (1) and rutin (2) in leaf sample replicates of injured susceptible soybeans.
**Figure S6.** Chromatogram of ions extraction of the flavonoid daidzin in leaf sample replicates of injured susceptible soybeans.
**Figure S7.** Chromatogram of ions extraction of the flavonoids hesperidin (1) and rutin (2) in leaf sample replicates of uninjured susceptible soybeans.
**Figure S8.** Chromatogram of ions extraction of the flavonoid daidzin in leaf sample replicates of uninjured susceptible soybeans.

## Data Availability

The data that support the results of the study are available from the corresponding author upon reasonable request.
