# Supplementary material for: Soybean induced resistance to Spodoptera eridania herbivory
Source: Pest Manag Sci. 2026 Jun 15;82(10):9931–43. doi: 10.1002/ps.71029 (PMC13569215; doi:10.1002/ps.71029)
Supplement: Supplementary file 1 [file PS-82-9931-s001.docx]

Supplementary figure 1. Chromatogram of ions extraction of the flavonoids hesperidin (1) and rutin (2) in leaf sample replicates of injured resistant soybean.

Supplementary figure 2. Chromatogram of ions extraction of the flavonoid isoquercitrin in leaf sample replicates of injured resistant soybean.

Supplementary figure 3. Chromatogram of ions extraction of the flavonoids hesperidin (1) and rutin (2) in leaf sample replicates of uninjured resistant soybean.

Supplementary figure 4. Chromatogram of ions extraction of the flavonoid isoquercitrin in leaf sample replicates of uninjured resistant soybean.

Supplementary figure 5. Chromatogram of ions extraction of the flavonoids hesperidin (1) and rutin (2) in leaf sample replicates of injured susceptible soybeans.

Supplementary figure 6. Chromatogram of ions extraction of the flavonoid daidzin in leaf sample replicates of injured susceptible soybeans.

Supplementary figure 7. Chromatogram of ions extraction of the flavonoids hesperidin (1) and rutin (2) in leaf sample replicates of uninjured susceptible soybeans.

Supplementary figure 8. Chromatogram of ions extraction of the flavonoid daidzin in leaf sample replicates of uninjured susceptible soybeans.
